# Wheelchair Basketball Competition Heart Rate Profile According to Players’ Functional Classification, Tournament Level, Game Type, Game Quarter and Playing Time

**DOI:** 10.3389/fpsyg.2019.00773

**Published:** 2019-04-15

**Authors:** Jolanta Marszałek, Karol Gryko, Andrzej Kosmol, Natalia Morgulec-Adamowicz, Anna Mróz, Bartosz Molik

**Affiliations:** ^1^Department of Rehabilitation, Józef Piłsudski University of Physical Education in Warsaw, Warsaw, Poland; ^2^Department of Physical Education, Józef Piłsudski University of Physical Education in Warsaw, Warsaw, Poland

**Keywords:** Paralympic sport, wheelchair basketball players, classification in sport, heart rate, match load, match analysis, physiological demands, adaptive sports

## Abstract

Heart rate is a popular parameter observed in team sports to plan training sessions with regard to load and sport specificity. Wheelchair basketball is an intermittent team game for physically impaired players. The study aim was to define heart rate profile of wheelchair basketball players in terms of their functional classification (category A: 1.0–2.5 points, category B: 3.0–4.5 points), tournament level (championships and friendly games), game type (close, balanced, and unbalanced), game quarter (1st, 2nd, 3rd, and 4th) and playing time (40–59%, 60–79%, and 80–100% in a quarter). Heart rate of 18 wheelchair basketball players was monitored in 22 games in four different tournaments, i.e., European Championships 2017, World Championships 2018, two friendly international tournaments of national teams (2017 and 2018). Heart rate (HR_mean_, HR_peak_, %HR_peak_, HRR, and %HRR) was monitored by Polar Team Pro (Kempele, Finland) during playing time on the court. Timeouts, quarter breaks, a half break, time on a bench were not taken into account in HR monitoring. The Kolmogorov–Smirnov test, the Mann–Whitney U test and the Kruskal–Wallis test were used. Fourteen players divided according to the classification into category A and B were included in the final calculations (n = 457 cases). Significantly higher HR_mean_, %HR_peak_, HR_peak_, and %HRR were noted among category B players, and higher %HR_peak_ and %HRR among category A players at the highest tournament level compared to friendly games. There were significant differences in %HRR and the percentage of time spent in HR zone I between the players with different playing time (40–59% versus 60–79%) in category B. No significant differences in HR were noted between four quarters. Among category A players, differences in HR in zone II were observed. Among category B players, statistically significant differences in % HR_peak_, the percentage of time spent in HR zones I, II, III, and %HRR between close, balanced and unbalanced games were found. In conclusion, the intermittent nature of wheelchair basketball was confirmed. Monitoring heart rate in a game could be helpful in creating exercises with proper loads for better physical preparation of wheelchair basketball players. High intensity training sessions would be more beneficial in preparing players for game demands.

## Introduction

Heart rate (HR) is a popular parameter showing the frequency of electrical heart activity in team sports training sessions and games. HR and oxygen consumption are used for predicting maximal oxygen consumption during tests. HR is a useful parameter to monitor exercise intensity, to assess fatigue status, and to quantify internal training loads in intermittent team sports, e.g., running basketball (Berkelmans et al., 2018).

Wheelchair basketball is an intermittent team game for people with physical impairments characterized by chronic or serious conditions limiting their possibility to use lower limbs to play running basketball, e.g., spinal cord injury, cerebral palsy, musculoskeletal conditions, spina bifida, amputation, poliomyelitis (Coutts, 1992; International Wheelchair Basketball Federation, 2014). The International Wheelchair Basketball Federation (IWBF) controls the classification and game rules of wheelchair basketball. Wheelchair basketball game rules are different compared to running basketball game rules due to the manner of moving (the player performs more than two pushes while in possession of the ball without dribbling, passing or shooting), type of faults (wheelchair contacts), and functional classification of players on the court (the sum of points of five players on the court cannot exceed 14.0 points, e.g., 4.5, 4.0, 3.0, 1.0, and 1.5) (International Wheelchair Basketball Federation, 2014; International Wheelchair Basketball Federation, 2018). All time rules (3 s in the opponents’ restricted area, 5 s to release the ball toward the court, 8 s to advance the ball over the center line, reset the shot clock to 14 s, 24 s to put up the legal shot, quarter time, break time), size of the court, the ball, height of baskets and point scoring system (throws for 1, 2, or 3 points) are the same for wheelchair basketball and running basketball (International Wheelchair Basketball Federation, 2018). Functional classification of the players is based on the observations carried out by experts (classifiers in wheelchair basketball) during a match. There are five main classes in wheelchair basketball: 1.0, 2.0, 3.0, 4.0, and 4.5, and three mixed classes 1.5, 2.5, and 3.5 (mixed functional characteristics of neighboring classes). Players are often divided into two categories: A (1.0–2.5) and B (3.0–4.5) (International Wheelchair Basketball Federation, 2014).

In wheelchair basketball, similar to running basketball, HR monitoring was applied to assess match load, i.e., the intensity of activity (Pérez et al., 2007; Croft et al., 2010; Yanci et al., 2014; Iturricastillo et al., 2016a,b; dos Santos et al., 2017). However, in wheelchair basketball it is important to underline certain disadvantages of HR measurement in people with spinal cord injury above Th5/6 characterized either by the loss of sympathetic outflow to the heart, where maximal HR (HR_peak_) is around 100 to 140 beats/min, or by autonomic dysreflexia (Theisen, 2012). Nevertheless, research related to running basketball is similar to research regarding wheelchair basketball. For instance, Delextrat and Kraiem (2013) showed that a small-sided game training exerted a positive influence on aerobic capacity and technical skills in running basketball players (Delextrat and Kraiem, 2013). Yanci et al. (2014) monitored HR during small-sided wheelchair basketball games (four sets in each session, 2-min intervals between the sets). They concluded that small-sided games are similarly demanding compared to official wheelchair basketball matches in terms of HR_mean_ values and can be a good predictive factor for a coach regarding the players’ anaerobic and aerobic preparation, and their reactions to the intensity of exercises (Yanci et al., 2014). Mason et al. (2018) compared 3 vs. 3 (small-sided games on a half court) and 5 vs. 5 wheelchair basketball games, and found that HR_peak_ and HR_mean_ were higher for 5 vs. 5 games. It turned out that 3 vs. 3 games are good to practice the specificity of wheelchair basketball because of more severe rotations, turnovers, rebounds and other high-intensity technical skills (Mason et al., 2018).

Taking into account all the above-mentioned studies (Pérez et al., 2007; Croft et al., 2010; Yanci et al., 2014; Iturricastillo et al., 2016a,b; dos Santos et al., 2017; Mason et al., 2018) the authors of only three of them compared the participants’ HR in a wheelchair basketball game according to the players’ functional classification (they were observing differences in HR between the players from category A and players from category B) (Pérez et al., 2007; Iturricastillo et al., 2016b; Marszalek et al., 2019). In three studies, HR, oxygen consumption and blood lactate were analyzed in an aerobic test and HR was monitored during an international wheelchair basketball competition (Croft et al., 2010; dos Santos et al., 2017; Marszalek et al., 2019). In other studies, HR during small-sided wheelchair basketball games was monitored (Yanci et al., 2014; Mason et al., 2018), and two methods of match load assessment, i.e., HR and rate of perceived exertion (RPE), were compared (Iturricastillo et al., 2016a). The research group in the above-mentioned studies included 3–10 wheelchair basketball players. Moreover, we found that there were no global analyses of players’ HR response and playing time, differences in HR between game quarters, tournament level, overall game outcome (final result of a game) regarding wheelchair basketball, but there was research on running basketball players (Vaquera Jiménez, 2008; Puente et al., 2017; Ramos-Campo et al., 2017; Montgomery and Maloney, 2018), and it also seems to be important for wheelchair basketball (Berkelmans et al., 2018). The importance of HR quantifying and monitoring with regard to the players’ classification as well as to playing time, game quarters, tournament level or game outcome would be useful for coaches and players to conduct training practice adapted to intermittent game effort (wheelchair basketball game load) and to plan pre-season and in-season exercise intensity for the players to reach the highest level according to their classification (type of impairments). Measuring HR according to playing time and game quarter will help coaches to create effective endurance training (to improve the players’ anaerobic and aerobic capacity). Taking into account these HR observations, coaches can adapt exercise time, number of repetitions and number of exercise series individually to each player. It will help coaches to conduct training sessions at the intensity similar to the one observed during a game (similar HR parameters of training efforts) with regard to the tournament level (championships or friendly games), the final game outcome (close, balanced, and unbalanced games), HR in a selected quarter (1st, 2nd 3rd, and 4th quarter) or playing time (40–59, 60–79, and 80–100% of playing time in a quarter). In particular, monitoring typical responses of players in a game allows basketball practitioners to better tailor training activities that meet or exceed the internal intensities of a game (Ben Abdelkrim et al., 2007). Thus, we see that these types of analyses would be helpful for wheelchair basketball coaches. The aim of the current study was to define the profile of heart rate of wheelchair basketball players in terms of their functional classification, tournament level, game type, game quarter and playing time.

## Materials and Methods

### Participants

Eighteen wheelchair basketball players (national team players) were monitored in four different tournaments, i.e., European Championships in Tenerife, Spain 2017, World Championships in Hamburg, Germany 2018 (eight and six games, respectively) and two friendly international tournaments of national teams (Walbrzych, Poland 2017 and 2018; four and four games, respectively). In total, 18 players in 22 games were observed and their HR was monitored. All the players were members of the national team. They practiced regularly (at least 6–8 h per week) with their league teams, and they participated in national camps: 7 days (1 week) per month in the 4th–6th month before the tournaments and 20 days per month in the 1st to 3rd month before the tournaments. Game schedule for European Championships in Tenerife, Spain (2017), and World Championships in Hamburg, Germany (2018) was similar (one game per day, in the afternoon or in the morning). During friendly international tournaments of national teams there were two games per day, one in the morning and one in the evening (with at least a 7-h break).

Participants were informed about the purpose of the study and were asked to sign the consent form. All the procedures were approved by the Local Bioethics Committees (the Commission of Ethics and Bioethics at Cardinal Stefan Wyszyński University in Warsaw: KEIB - 10/2016, and the Senate Ethics Commission at Jozef Piłsudski University of Physical Education in Warsaw: SKE 01-16/2017) and were completed in accordance with the ethical standards as described in the Declaration of Helsinki.

Playing time in a quarter (a player had to spend at least 40% of the time in a quarter without timeouts or substitutions; at least 6 min in a quarter) was an inclusion criterion applied in the study. For instance, one player was on the court for the first 6 min in the first quarter. A coach took timeout and the same player participated in this quarter for the next 5 min. The first quarter took 15 min in total (with breaks, timeouts, etc.) and the athlete’s playing time in a quarter was 40 and 33%, respectively. In our analysis, we included the 6 min of his playing time in a quarter because it took more than 40% of the total time of the first quarter. Moreover, the player had to play at least five times on the court in one match. In conclusion, any problem with a chest strap, less than 40% of playing time in a quarter without time outs or substitutions, less than 5 times on the court in one match excluded the player from our calculations. The inclusion and exclusion criteria concerned each participant in each game type (close, balanced, unbalanced, championships, and friendly games).

### Heart Rate Recording

Heart rate (HR; HR_mean_, HR_peak_, and %HR_peak_) of all the players was monitored during each match with the use of downloadable, wireless Polar Team Pro (Polar Team Pro, Kempele, Finland) and Polar heart rate sensor attached to a chest strap. HR frequency was coded at 1-s registration intervals. Inactive time (e.g., quarter breaks, half-time break, time outs in each match) was also registered by Polar Team Pro. Each situation in a game such as the start of a match, quarter breaks, a half-time break, time outs in each match, substitutions of each player, time after the end of a match was marked in the Polar Team Pro software. Moreover, markers applied in the Polar Team Pro software and observations of all the matches on video footage made it possible to delete the time of the above-mentioned situations from the players’ HR history.

Five HR zones (I – 50–59%, II – 60–69%, III – 70–79%, IV – 80–89%, and V – 90–100%) are originally set in the Polar Team Pro software and % of time in each zone was taken as outcome measure. Heart rate reserve (HRR) and the percentage of heart rate reserve (%HRR) were calculated. HRR is the difference between maximum heart rate (HR_peak_) from a valid and reliable aerobic test and resting heart rate (HR_rest_) (Janssen et al., 1994; Pérez et al., 2003):

[1] HRR = HR_peak_ from a test – HR_rest_

The percentage of heart rate reserve (%HRR) was calculated using the formula:

[2] %HRR = (HR_mean_ – HR_rest_)^∗^100/HRR

To do the above calculations, HR_peak_ for each player was determined before match analyses (1–4 weeks before tournaments, May 2017 and May 2018) in a valid and reliable aerobic performance laboratory test (Molik et al., 2017; Marszalek et al., 2019) on an arm crank ergometer (Lode ACE; Groningen; Netherlands). Resting heart rate (HR_rest_) was measured in each player on the examination day in the morning immediately after waking up.

### Analyzed Variables

Heart rate profile of wheelchair basketball players was observed in terms of their functional classification, tournament level, game type, game quarter, and playing time.

Functional classification of all the players was done by IWBF panel of classifiers, and the players were divided into two functional categories: A and B (category A players with the classification of 1.0–2.5 points and category B players with the classification of 3.0–4.5 points). Tournament level means that the analyzed games were divided into championships (European Championships 2017 and World Championships 2018) and friendly international games (two friendly international tournaments). Playing time means that a player spent 40–59, 60–79, or 80–100% of the time playing actively in a game. HR was also analyzed with regard to a quarter (1st, 2nd, 3rd, and 4th quarter), and final point differential (close games – differences in the scores ranged from 1 to 6 points, balanced games – differences in the scores ranged from 7 to 17 points, and unbalanced games - differences in the scores were larger than 18 points).

### Statistical Analysis

All the analyses were performed using the SPSS IBM Statistics 24 for Windows. Means and standard deviation (SD) of HR data were calculated. The distribution of the results was checked with the use of the Kolmogorov–Smirnov test. All the results had non-parametric distribution so the Mann–Whitney *U* test and the Kruskal–Wallis test for independent samples were used to compare the results of players depending on their functional category (category A and B), tournament level (European Championships 2017 and World Championships 2018 versus two friendly international tournaments), playing time (40–59, 60–79, and 80–100%), quarter (1st, 2nd, 3rd, and 4th quarter), and final point differential (the cluster analysis showed the following: close games – differences in the scores ranged from 1 to 6 points, balanced games – differences in the scores ranged from 7 to 17 points and unbalanced games – differences in the scores were larger than 18 points). The significance level deemed acceptable was *p* < 0.05. Additionally, effect size (ES) was calculated. The following levels of effect sizes were estimated: small 0.2, medium 0.5, and large 0.8 (Cohen, 1988).

## Results

After considering all inclusion and exclusion criteria, 14 out of 18 players were taken into account in the final calculations and we received 457 cases (HR data) to analyze. The characteristics of HR data of wheelchair basketball players are presented in Table 1. These 14 players were divided into two groups, i.e., category A (functional classification 1.0–2.5 points; *n* = 6) and category B (functional classification 3.0–4.5 points; *n* = 8). Category A players had such impairments as spinal cord injury (*n* = 6) and category B players had such impairments as amputation (*n* = 4), spina bifida (*n* = 2), cerebral palsy (*n* = 1), and others (*n = 1)*.

**Table 1:** The characteristics of heart rate data of wheelchair basketball players (*N* = 457 cases).

Parameters	Median	Mean	*SD*
HR_mean_ [beats/min]	159.00	156.78	15.69
HR_peak_ [beats/min]	177.00	174.52	13.29
HR_peak_ [%]	96.00	96.48	6.48
HR zone I [%]	0.00	0.85	3.75
HR zone II [%]	1.06	5.25	11.04
HR zone III [%]	10.41	15.37	15.55
HR zone IV [%]	37.47	36.91	20.35
HR zone V [%]	38.21	38.70	27.83
HRR	124.00	121.90	9.10
%HRR	79.84	78.93	10.23

*N, number of cases; HR, heart rate; SD, standard deviation; HRmean, mean heart rate; %HRpeak, the percentage of maximal heart rate in a match; HRpeak, maximal heart rate in a match; HRR, heart rate reserve; %HRR, the percentage of heart rate reserve.*

Table 2 shows differences in the analyzed HR data of wheelchair basketball players between the two functional categories (A and B). Significantly higher values of HR_mean_, HR_peak_, %HR_peak_, HRR and %HRR were observed in players from category B, while the effect size was large. Players from category B spent significantly more time in HR zone V compared to category A players (43.24% vs. 31.12%). There were no differences in the percentage of time spent in HR zone IV between the players from category A and B (Table 2).

**Table 2:** Differences in heart rate data of wheelchair basketball players according to functional category (A and B).

	Wheelchair basketball players													
Parameters	Category A (class from 1.0 to 2.5) (*n* = 6)				Category B (class from 3.0 to 4.5) (*n* = 8)						
	Median	Mean	*SD*	*N*	Median	Mean	*SD*	*N*	*Z*	*p*	ES
HR_mean_ [beats/min]	151.00	148.99	17.48	171	162.00	161.44	12.39	286	−7.297	^∗∗∗^	2.49
HR_peak_ [beats/min]	171.00	167.81	15.21	171	180.00	178.53	10.06	286	−7.436	^∗∗∗^	2.59
HR_peak_ [%]	95.00	94.51	6.43	171	97.00	97.66	6.22	286	−4.000	^∗∗∗^	0.75
HR zone I [%]	0.00	1.67	5.81	171	0.00	0.35	1.33	286	−3.416	^∗∗∗^	0.55
HR zone II [%]	2.27	8.96	15.31	171	0.25	3.03	6.47	286	−5.044	^∗∗∗^	1.19
HR zone III [%]	16.15	19.75	17.52	171	8.73	12.75	13.62	286	−4.203	^∗∗∗^	0.83
HR zone IV [%]	39.55	37.53	18.66	171	35.45	36.54	21.32	286	−0.862	n.s.	−
HR zone V [%]	27.44	31.12	26.96	171	43.90	43.24	27.40	286	−4.570	^∗∗∗^	0.98
HRR	119.00	117.99	8.39	171	124.00	124.23	8.71	286	−7.491	^∗∗∗^	2.62
%HRR	76.85	75.37	11.64	171	80.69	81.06	8.64	286	−5.160	^∗∗∗^	1.25

*^∗∗∗^p < 0.001; n.s., no statistically significant differences; N, number of cases; HR, heart rate; SD, standard deviation; Min, minimum; Max, Maximum; ES, effect size for the U-Mann–Whitney test; HRmean, mean heart rate; %HRpeak, the percentage of maximal heart rate in a match; HRpeak, maximal heart rate in a match; HRR, heart rate reserve; %HRR, the percentage of heart rate reserve.*

Table 3 shows differences in the analyzed HR data separately for wheelchair basketball players from category A and B according to the tournament level (European Championships 2017 and World Championships 2018 versus two friendly international tournaments). Significantly higher HR_mean_, %HR_peak_, HR_peak_, and %HRR were observed in the players from category B in the highest level of tournaments compared to friendly games. The effect size was large or medium for these differences. The values of %HR_peak_ and %HRR in players from category A were significantly higher in the highest-level tournaments compared to friendly games (Table 3).

**Table 3:** Differences in heart rate data of wheelchair basketball players from category A and B according to tournament level.

	Championships				Friendly international tournaments						
Parameters	Median	Mean	*SD*	*N*	Median	Mean	*SD*	*N*	*Z*	*p*	ES
**Wheelchair basketball players category A**											
HR_mean_ [beats/min]	152.00	151.26	16.03	100	151.00	145.79	19.00	71	−1.604	n.s.	−
HR_peak_ [beats/min]	170.00	169.43	14.03	100	172.00	165.52	16.56	71	−1.315	n.s.	−
HR_peak_ [%]	96.00	95.96	5.72	100	93.00	92.46	6.85	71	−3.711	^∗∗∗^	1.05
HR zone I [%]	0.00	0.55	1.77	100	0.00	3.24	8.55	71	−2.331	^∗^	0.42
HR zone II [%]	1.28	6.84	13.60	100	4.55	11.95	17.09	71	−2.885	^∗∗^	0.64
HR zone III [%]	11.19	16.33	15.59	100	20.16	24.57	19.02	71	−2.938	^∗∗^	0.66
HR zone IV [%]	39.33	38.42	17.85	100	39.61	36.28	19.80	71	−0.513	n.s.	−
HR zone V [%]	35.93	36.54	26.56	100	16.32	23.48	25.81	71	−3.379	^∗∗∗^	0.87
HRR	111.00	116.36	7.77	100	123.00	120.28	8.75	71	−2.950	^∗∗^	0.67
%HRR	80.47	78.72	10.82	100	71.20	70.64	11.17	71	−4.406	^∗∗∗^	0.48
**Wheelchair basketball players category B**											
HR_mean_ [beats/min]	165.00	164.38	11.98	180	157.00	156.45	11.49	106	−5.143	^∗∗∗^	1.56
HR_peak_ [beats/min]	181.00	180.63	9.66	180	176.00	174.95	9.75	106	−4.529	^∗∗∗^	1.21
HR_peak_ [%]	97.00	98.24	5.69	180	95.00	96.68	6.96	106	−2.467	^∗^	0.36
HR zone I [%]	0.00	0.28	1.16	180	0.00	0.48	1.58	106	−2.944	^∗∗^	0.51
HR zone II [%]	0.00	1.95	4.26	180	1.72	4.87	8.79	106	−4.465	^∗∗∗^	1.18
HR zone III [%]	7.89	10.57	11.76	180	12.80	16.46	15.68	106	−3.413	^∗∗∗^	0.69
HR zone IV [%]	30.67	34.20	20.55	180	41.92	40.51	22.11	106	−2.329	^∗^	0.32
HR zone V [%]	51.48	48.93	26.51	180	33.51	33.58	26.28	106	−4.572	^∗∗∗^	1.24
HRR	124.00	124.81	8.80	180	124.00	123.25	8.50	106	−1.414	n.s.	−
%HRR	82.74	83.19	8.43	180	78.08	77.44	7.77	106	−3.394	^∗∗∗^	1.72

*^∗^p < 0.05; ^∗∗^p < 0.01; ^∗∗∗^p < 0.001; n.s., no statistically significant differences; N, number of cases; HR, heart rate; SD, standard deviation; Min, minimum; Max, Maximum; ES, effect size for the U-Mann–Whitney test; HRmean, mean heart rate; %HRpeak, the percentage of maximal heart rate in a match; HRpeak, maximal heart rate in a match; HRR, heart rate reserve; %HRR, the percentage of heart rate reserve.*

Table 4 shows differences in HR data of wheelchair basketball players from category A and B according to playing time. There were no statistically significant differences in HR data between the percentage of playing time for category A players. Two differences in %HRR and the percentage of time spent in HR zone I were found with regard to playing time (40–59% versus 60–79%; Table 4) for players from category B (*n* = 8).

**Table 4:** Differences in heart rate data of wheelchair basketball players from category A and B according to playing time.

	Players with 40–59% of playing time				Players with 60–79% of playing time				Players with 80–100% of playing time					
Parameters	Median	Mean	*SD*	*N*	Median	Mean	*SD*	*N*	Median	Mean	*SD*	*N*	*p*	ES
**Wheelchair basketball players category A**														
HR_mean_ [beats/min]	153.00	149.47	17.08	99	147.00	146.58	19.36	33	151.00	149.79	17.12	39	n.s.	−
HR_peak_ [beats/min]	171.00	167.77	15.30	99	167.00	165.82	15.87	33	170.00	169.59	14.59	39	n.s.	−
HR_peak_ [%]	95.00	94.40	6.93	99	94.00	93.73	6.63	33	96.00	95.44	4.76	39	n.s.	−
HR zone I [%]	0.00	1.54	5.80	99	0.00	2.86	8.00	33	0.00	0.99	2.90	39	n.s.	−
HR zone II [%]	2.34	9.17	16.57	99	2.63	9.58	14.62	33	2.05	7.91	12.61	39	n.s.	−
HR zone III [%]	16.91	19.64	17.73	99	19.60	22.60	18.49	33	12.31	17.60	16.23	39	n.s.	−
HR zone IV [%]	39.00	36.29	18.32	99	41.51	37.54	22.34	33	41.19	40.68	16.04	39	n.s.	−
HR zone V [%]	30.35	32.43	28.33	99	21.20	25.55	25.57	33	29.83	32.52	24.42	39	n.s.	−
HRR	123.00	118.40	8.39	99	111.00	116.36	7.99	33	111.00	118.33	8.76	39	n.s.	−
%HRR	76.42	75.49	11.52	99	76.42	74.85	13.72	33	77.48	75.48	10.26	39	n.s.	−
**Wheelchair basketball players category B**														
HR_mean_ [beats/min]	164.00	162.30	12.44	150	158.00	158.64	12.69	75	162.00	162.79	11.53	61	n.s.	−
HR_peak_ [beats/min]	180.00	178.79	9.62	150	177.00	176.73	10.67	75	180.00	180.10	10.17	61	n.s.	−
HR_peak_ [%]	96.00	97.69	5.97	150	96.00	96.97	6.43	75	97.00	98.46	6.56	61	n.s.	−
HR zone I [%]	0.00	0.31	1.33	150^∧^	0.00	0.48	1.64	75^∧^	0.00	0.31	0.83	61	^∗^	0.52
HR zone II [%]	0.00	3.17	7.72	150	0.69	3.67	5.72	75	0.79	1.91	2.97	61	n.s.	−
HR zone III [%]	8.47	11.75	12.34	150	10.42	15.43	15.02	75	8.21	11.94	14.57	61	n.s.	−
HR zone IV [%]	30.52	35.07	21.78	150	39.87	38.70	19.08	75	41.12	37.50	22.82	61	n.s.	−
HR zone V [%]	46.61	45.11	26.83	150	39.62	39.66	28.76	75	43.80	43.02	27.11	61	n.s.	−
HRR	124.00	123.52	8.55	150	124.00	124.89	9.10	75	124.00	125.18	8.62	61	n.s.	−
%HRR	82.24	82.22	8.96	150^∧^	78.23	78.47	8.32	75^∧^	80.15	81.40	7.61	61	^∗^	0.77

*^∗^p < 0.05; ^∧^, differences between groups p < 0.05; n.s., no statistically significant differences; N, number of cases; HR, heart rate; SD, standard deviation; Min, minimum; Max, Maximum; ES, effect size for the U-Mann–Whitney test; HRmean, mean heart rate; %HRpeak, the percentage of maximal heart rate in a match; HRpeak, maximal heart rate in a match; HRR, heart rate reserve; %HRR,the percentage of heart rate reserve.*

Table 5 shows differences in HR data of wheelchair basketball players from category A and B with regard to game quarter. We did not observe statistically significant differences in any HR data of players from category A and B between four quarters (Table 5).

**Table 5:** Differences in heart rate data of wheelchair basketball players from category A and B according to game quarter.

	1st quarter				2nd quarter				3rd quarter				4th quarter				
Parameters	Median	Mean	*SD*	*N*	Median	Mean	*SD*	*N*	Median	Mean	*SD*	*N*	Median	Mean	*SD*	*N*	*p*
**Wheelchair basketball players category A**																	
HR_mean_ [beats/min]	150.50	149.81	17.92	48	154.00	149.24	18.43	46	150.00	146.72	15.92	43	155.00	150.35	17.94	34	n.s.
HR_peak_ [beats/min]	168.00	168.13	15.70	48	172.50	167.78	16.43	46	169.00	166.26	14.16	43	171.00	169.35	14.55	34	n.s.
HR_peak_ [%]	96.00	95.21	6.33	48	95.00	94.46	7.16	46	94.00	93.51	6.06	43	95.00	94.85	6.08	34	n.s.
HR zone I [%]	0.00	1.75	5.00	48	0.00	1.60	5.32	46	0.00	1.92	7.28	43	0.00	1.34	5.65	34	n.s.
HR zone II [%]	1.84	8.22	13.60	48	2.18	10.04	18.53	46	2.95	8.34	12.74	43	2.31	9.34	16.32	34	n.s.
HR zone III [%]	9.28	15.52	15.95	48	18.37	20.13	17.48	46	19.70	24.81	19.74	43	16.36	18.80	15.71	34	n.s.
HR zone IV [%]	37.96	38.71	19.72	48	32.59	33.86	18.63	46	44.37	40.64	17.86	43	37.63	36.89	18.05	34	n.s.
HR zone V [%]	32.80	35.16	26.84	48	24.67	32.54	29.77	46	15.89	23.77	24.52	43	30.49	32.79	25.38	34	n.s.
HRR	111.00	117.19	8.31	48	119.00	117.91	7.99	46	119.00	118.67	8.44	43	115.00	118.35	9.45	34	n.s.
%HRR	78.39	76.12	11.11	48	77.64	75.89	12.43	46	74.07	73.28	10.60	43	78.51	76.25	12.26	34	n.s.
**Wheelchair basketball players category B**																	
HR_mean_ [beats/min]	164.00	162.32	10.88	82	160.00	161.39	12.50	74	159.00	159.16	12.08	67	165.00	162.79	14.26	63	n.s.
HR_peak_ [beats/min]	178.00	178.24	9.74	82	180.00	179.30	9.46	74	176.00	176.94	9.48	67	181.00	179.68	11.63	63	n.s.
HR_peak_ [%]	97.00	97.83	6.28	82	98.00	98.07	6.20	74	96.00	97.01	6.37	67	96.00	97.67	6.11	63	n.s.
HR zone I [%]	0.00	0.30	0.80	82	0.00	0.47	1.62	74	0.00	0.44	1.84	67	0.00	0.19	0.75	63	n.s.
HR zone II [%]	0.76	2.09	3.60	82	0.00	3.79	8.78	74	0.65	3.26	6.30	67	0.00	3.13	6.37	63	n.s.
HR zone III [%]	6.44	10.24	12.80	82	9.87	12.70	12.12	74	9.68	14.84	14.43	67	9.00	13.87	15.17	63	n.s.
HR zone IV [%]	36.68	38.19	22.89	82	29.77	33.35	20.27	74	40.87	40.58	21.54	67	31.94	33.83	19.67	63	n.s.
HR zone V [%]	45.28	44.72	27.02	82	48.55	46.09	28.03	74	39.88	37.26	27.11	67	47.31	44.30	27.18	63	n.s.
HRR	124.00	124.63	9.38	82	124.00	123.46	9.01	74	124.00	124.21	8.70	67	124.00	124.66	8.93	63	n.s.
%HRR	80.88	81.56	7.06	82	80.19	81.56	9.11	74	80.15	79.21	8.22	67	81.45	81.79	10.18	63	n.s.

*^∗^p < 0.05; n.s., no statistically significant differences; N, number of cases; HR, heart rate; SD, standard deviation; Min, minimum; Max, Maximum; ES, effect size for the U-Mann–Whitney test; HRmean, mean heart rate; %HRpeak, the percentage of maximal heart rate in a match; HRpeak, maximal heart rate in a match; HRR, heart rate reserve; %HRR,the percentage of heart rate reserve.*

Table 6 shows differences in HR data of wheelchair basketball players from category A and B according to final point differential (game types: close, balanced and unbalanced games). Among players from category A, no differences in HR data were noted except for the percentage of time spent in HR zone IV and HRR between balanced and unbalanced games, and HRR between close and unbalanced games (in each case, higher values of variables were observed in unbalanced games). In the case of category B players, there were statistically significant differences between close and balanced games regarding HR_peak_ (higher values were observed in balanced games), between the percentage of time spent in HR zones IV (higher values were observed in close games) and V (higher values were observed in balanced games), between close and unbalanced games in HRR (higher values were observed in close games), and between balanced and unbalanced games in HR_peak_ and %HR_peak_ (higher values were observed in balanced games)_,_ as well as in the percentage of time spent in HR zones III and IV (higher values were observed in unbalanced games) and in zone V (higher values were observed in balanced games) and HRR (higher values were observed in balanced games) (Table 6).

**Table 6:** Differences in heart rate data of wheelchair basketball players from category A and B according to final point differential.

	Close games Score difference between 1–6 points (*n* = 8)				Balanced games Score difference between 7–17 points (*n* = 10)				Unbalanced games Score difference above 18 points (*n* = 5)					
Parameters	Median	Mean	*SD*	*N*	Median	Mean	*SD*	*N*	Median	Mean	*SD*	*N*	*p*	ES
**Wheelchair basketball players category A**														
HR_mean_ [beats/min]	150.00	147.42	19.31	72	147.00	147.96	17.33	53	155.50	152.63	14.15	46	n.s.	−
HR_peak_ [beats/min]	167.50	165.82	16.66	72	169.00	167.43	14.68	53	175.00	171.35	12.96	46	n.s.	−
HR_peak_ [%]	95.00	93.50	7.30	72	96.00	95.49	5.93	53	96.00	94.96	5.33	46	n.s.	−
HR zone I [%]	0.00	2.91	8.52	72	0.00	0.96	2.31	53	0.00	0.55	1.56	46	n.s.	−
HR zone II [%]	2.35	9.61	15.59	72	2.56	9.86	14.85	53	1.92	6.93	15.55	46	n.s.	−
HR zone III [%]	18.66	21.85	18.82	72	15.62	19.76	18.50	53	12.47	16.45	13.69	46	n.s.	−
HR zone IV [%]	39.81	36.84	21.09	72	30.95	32.60	15.02	53§	46.21	44.30	16.61	46§	^∗∗^	1.18
HR zone V [%]	17.07	27.42	27.85	72	34.29	35.79	28.00	53	28.69	31.53	23.82	46	n.s.	−
HRR	119.00	118.00	8.64	72 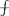	111.00	115.26	7.31	53§	125.00	121.11	8.24	46 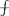 §	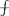 ^∗^ §^∗∗^	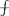 0.38 §1.07
%HRR	74.89	74.12	13.05	72	79.28	76.91	11.66	53	76.64	75.54	8.98	46	n.s.	−
**Wheelchair basketball players category B**														
HR_mean_ [beats/min]	162.50	160.90	11.56	108	164.00	162.98	12.31	104	159.00	160.08	13.57	74	n.s.	−
HR_peak_ [beats/min]	179.00	177.42	9.52	108^∧^	181.00	180.64	9.66	104^∧^§	176.50	177.18	10.98	74§	^∧∗^ §^∗^	^∧^0.39 §0.44
HR_peak_ [%]	96.00	97.72	6.91	108	98.00	98.91	5.70	104§	96.00	95.82	5.45	74§	§^∗∗∗^	§0.99
HR zone I [%]	0.00	0.20	0.67	108	0.00	0.26	1.05	104	0.00	0.71	2.13	74	n.s.	−
HR zone II [%]	0.00	2.85	5.15	108	0.06	2.95	7.98	104	1.51	3.43	5.88	74	n.s.	−
HR zone III [%]	8.01	12.49	14.55	108	8.04	10.90	11.77	104§	12.50	15.74	14.29	74§	^∗^	0.32
HR zone IV [%]	39.36	38.51	23.78	108^∧^	29.25	31.03	17.75	104^∧^§	42.61	41.41	20.71	74§	^∧∗^ §^∗∗∗^	^∧^0.33 §0.81
HR zone V [%]	37.97	40.12	28.17	108^∧^	52.06	51.17	24.73	104^∧^§	34.84	36.63	27.53	74§	^∧∗∗^ §^∗∗∗^	^∧^0.56 §0.88
HRR	124.00	124.64	8.58	108 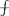	124.00	125.63	8.63	104§	121.00	121.68	8.58	74 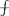 §	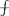 ^∗^ §^∗∗^	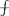 0.47 §0.72
%HRR	80.70	80.53	7.66	108	81.54	81.67	8.50	104	79.63	80.99	10.12	74	n.s.	−

*^∗^p < 0.05, ^∗∗^p < 0.01, ^∗∗∗^p < 0.001; ^∧^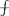 §, differences between groups p < 0.05; n.s., no statistically significant differences; N, number of cases; HR, heart rate; SD, standard deviation; Min, minimum; Max, maximum; ES, effect size for the U-Mann–Whitney test; HRmean, mean heart rate; %HRpeak, the percentage of maximal heart rate in a match; HRpeak, maximal heart rate in a match; %HRR, the percentage of heart rate reserve.*

## Discussion

The aim of this study was to define the profile of heart rate of wheelchair basketball players in terms of their functional classification, tournament level, game type, game quarter and playing time.

Considering all exclusion and inclusion criteria, HR data of 14 people were analyzed. The players’ HRR, HR_mean_, HR_peak_, %HR_peak_ were high and %HRR was 78.9%, which demonstrates the demanding nature of wheelchair basketball (Janssen et al., 1994; Pérez et al., 2007). This information can be a premise for physical performance coaches to determine HRR zones in which each wheelchair basketball player should train to be well prepared to basketball games, regardless of tournament rank or match score. HRR zones should be determined individually for each player considering their HR_rest_, HR_peak_, which was also underlined by Croft et al. (2010). It should be highlighted that our study results as well as the findings of Croft et al. (2010) indicated that high-intensity training sessions may be more beneficial for wheelchair basketball players and better prepare players for games (Croft et al., 2010).

While comparing our results to the results of other studies, we received similar values of %HRR to the ones obtained by Pérez et al. (2007), i.e., 78.9 and 78.5%, respectively (Pérez et al., 2007). However, we expected to observe differences in %HRR between players from the category A and B. Therefore, we divided our research group and finally confirmed these differences in %HRR (75.4% vs. 81.1%; *p* < 0.001). Pérez et al. (2007) evaluated five participants (highly trained wheelchair basketball players), divided according to their functional classification (two players from category A, and three players from category B) and found that category A players manifested lower HR_mean_ and %HRR compared to category B (143.9 and 73.1% vs. 162.7 and 81.7%, respectively) (Pérez et al., 2007). In this study we observed significantly higher HR_mean_ and similar %HRR compared to the results of Pérez et al. (2007) (HR_mean_: category A players – 167.8 vs. 143.9 and category B players – 178.5 vs. 162.7; %HRR: category A players – 75.4% vs. 73.1% and category B players – 81.1% vs. 81.7%). These results indicate that the participants of our study achieved higher HR_peak_ or lower HR_rest_ (the formula of %HRR). However, in our study more participants were recruited than in the study by Pérez et al. (2007) and a different protocol of a laboratory test to access HR_peak_ was used (Pérez et al., 2007).

We found one study (a case study) comparing HR response of a player with spinal cord injury (level TH 1–2) with two players without spinal cord injury (Iturricastillo et al., 2016b). The authors of this study observed higher HR_peak_ and HR_mean_ in the players without spinal cord injury. They underlined that the autonomic innervation of the heart influences HR_peak_ but relative HR (%HR_peak_) can be similar in these two groups (Iturricastillo et al., 2016b). Moreover, we considered the explanation by dos Santos et al. (2017) that less trained players will experience more physiological stress in a match (dos Santos et al., 2017). therefore, in order to avoid this aspect in our study, we selected well prepared (in terms of aerobic and anaerobic performance) elite players from the national team who won sixth place in the European Championships in 2017 and sixth place in the World Championships in 2018. We concluded that the observed HR data in the current study support wheelchair basketball as a demanding team game that comprises of short efforts like dynamic pushing and breaking, passes, turnovers, shooting, etc., which is reflected in high HRR, HR_mean_, HR_peak_, %HR_peak_.

We aimed at confirming the hypothesis that players with spinal cord injury are characterized by higher HR data (Iturricastillo et al., 2016b). In the current study, we divided players into two functional groups, i.e., category A (players with a spinal cord injury) and category B (players without a spinal cord injury), to analyze their results separately. In our opinion, if it is possible, it should be a recommendation for all future studies on wheelchair basketball to divide participants at least into two functional categories because of the specificity of wheelchair basketball rules and classification. In the current study, we noted statistically significant differences in all HR data between category A and B. Differences in HR data between these two categories were also confirmed in the previous analysis of other authors (Pérez et al., 2007; Marszalek et al., 2019). However, in the current study we divided players into groups and observed their playing time (time without the half break, time outs, and time staying on the bench), which seems to be a more proper approach to the match load assessment. This approach was also underlined by Marszalek et al. (2019), as they examined the players’ HR taking into account their playing time together with all breaks, stops, etc., and they suggested observing HR data in a game without a half break, timeouts, other breaks in the future studies because the results of HR_mean_ compared to HR_peak_ were low (for category A players the results were 120 beats/minute and 174 beats/minute, respectively; for category B players it was136 beats/minute and 183 beats/minute, respectively), and these breaks influenced HR_mean_ (Marszalek et al., 2019).

In the current study, it was observed that the percentage of time spent in HR zones (except in zone IV) differed between category A and category B players, and both groups spent the longest time in zones IV and V. Summarizing these differences regarding time contribution in HR zones between the players with and without spinal cord injury, it may be concluded that differences occurred due to the impairment, as players from category A could not achieve HR zone V for a long time (players from category B were significantly longer in HR zone V; large effect size: ES = 1.61) and their %HR_peak_ was also significantly lower compared to the players from category B (large effect size: ES = 1.23). This finding is opposite to the one presented by Iturricastillo et al. (2016a) that relative HR parameters (%HR_peak_) and the percentage of time spent in a high-intensity zone (85–95% of HR_peak_) was similar among their players. Their finding indicated that it was not the impairment, but probably the characteristics of a basketball game (tactical and strategic aspects, position of a player) that influences the percentage of time spent in HR zones (Iturricastillo et al., 2016a).

Pérez et al. (2007) also highlighted the fact that some situations during a basketball game are more demanding than others. They introduced a categorical frame of observation and established seven different “game categories” (Pérez et al., 2007). An offensive game with the ball was the most demanding part of a match (high HR data), which is the confirmation of previous analyses of Coutts (1992) and Gomez et al. (2015) (Coutts, 1992; Gomez et al., 2015). It was shown that high-point players (category B) had a much bigger number of game actions with the ball (shooting, rebounding, and stills) compared to low-point players (category A) (Vanlandewijck et al., 2003; Molik et al., 2009; Gomez et al., 2015). In the current study, all the players from category A had spinal cord injury below TH6 and they used wheelchairs in everyday life. Players from category B were at least able to stand, which means that lower limbs had muscle power (no significant atrophy or vascular tissues) and were active in terms of isometric strength in wheelchair propulsion, and their cardiovascular system was active in the whole body (Theisen and Vanlandewijck, 2002). It is an additional argument that the impairment may be the cause of HR data differences. However, Baumgart et al. (2018) suggested that disability possibly influences VO_2peak_ in Paralympic sitting sports (a high variation in VO_2peak_) with different disability classes compared to disciplines without different sports classes and recommended further research (Baumgart et al., 2018).

In the second part of this study, we noticed that all HR data (except HRR) were significantly different during Championships matches compared to friendly tournament matches for players from category B. Less significant differences were observed for players from category A (HR_mean_ and HR_peak_ were not different). Different HR data in different tournament rank competitions were noticed probably because of players’ higher motivation and excitement as well as better physical preparation during Championships matches (aerobic capacity and anaerobic performance) to the high-level competition. We underline that it is only a hypothesis because no research has been conducted examining this. Iturricastillo et al. (2018) observed higher intensity during playoffs compared to friendly wheelchair basketball games (higher HR_mean_ and HR_peak_) (Iturricastillo et al., 2018). However, Montgomery et al. (2010) compared HR between training games (5 on 5 scrimmage) and tournament games in running basketball and found that HR_mean_ and HR_peak_ were similar (Montgomery et al., 2010). These comparisons seem to be interesting and they show that even though wheelchair basketball has similar game rules to running basketball and both games are dynamic and intermittent, such situations as friendly games, scrimmages or small-sided games on a half court require a different response from wheelchair basketball players than from running basketball players.

In the third part of our study, we wanted to compare HR between the players from category A and B according to their playing time. We divided the players into three sub-groups in terms of their percentage contribution to playing time in a game: 40–59, 60–79, and 80–100%. There were no statically significant differences in HR data between the players from category A. These results indicate that players were engaged similarly and could maintain a similar level of intensity regardless of time which they spent on the court in a match. Significantly higher %HRR values were observed among category B players whose playing time was at the level of 40–59% than among the players whose contribution was at the level of 60–79%. We suppose that players who spend less time on the court in a quarter (lower percentage contribution in playing time) can achieve higher-intensity effort (higher %HRR because they are less tired). For coaches it can be an important lead to substitute players from category B every 6 min while the quarter takes around 15 min (40% of playing time in a quarter). On the other hand, Iturricastillo et al. (2018) also checked differences in HR data in terms of the participants’ playing time, but did not remove the time of breaks and timeouts from the calculation. They observed HR data with all breaks in a game and they found significant differences in HR_mean_ and HR_peak_ which were higher in athletes who played between 30 and 40 min compared to those who played shorter (Iturricastillo et al., 2018). In other words, players with more bench time will have lower HR_mean_ and HR_peak_ if break times are included in HR game analyses. Breaks and active time on the court can be taken into account in global HR analysis to see the ratio between breaks and active time on the court. This approach could provide some direct indication of training based on the ratio of breaks and active time of exercises.

In the fourth part of this study, we found that there were no significant differences in HR data of players from category A and B between all four quarters in wheelchair basketball. However, it is quite easy to observe the trend that the first and the last quarters are the most important in wheelchair basketball because %HRR was the highest in these quarters (however, these differences were not statistically significant). The analysis of HR in different parts of a wheelchair basketball match was conducted by Yanci et al. (2014), who monitored HR during small-sided wheelchair basketball games (Yanci et al., 2014). They divided a small-sided game into four bouts, and found significant differences regarding HR_peak_ and HR_mean_ between bouts 2, 3, 4 and bout 1 (HR_peak_ and HR_mean_ were significantly lower in bout 1). Further analyses are necessary to explore this aspect in more detail because the study by Yanci et al. (2014) delivered different findings to our study. Yanci et al. (2014) found that HR_peak_ and HR_mean_ in the first bout were significantly the lowest (Yanci et al., 2014). In our study we observed that HR_peak_ and HR_mean_ in all quarters were similar and the highest values were noticed in the first and the last quarter.

In the last part of this study, we noted similar values of HR data among players from category A related to final point differential (close, balanced, and unbalanced games). However, significant differences in HR data were noted among the players from category B between close and balanced games as well as between close and unbalanced games. In close and balanced games HR_peak_ and the percentage of time spent in HR zone V were significantly higher probably because of the fact that players from category B had more contact with the ball. Coutts (1992) and Pérez et al. (2007) wrote that offensive game with the ball was the most demanding part of a match, Gomez et al. (2015) evidenced that players from category B have more contact with the ball and Gomez et al. (2014) observed that they shoot successfully more often in balanced games (Coutts, 1992; Pérez et al., 2007; Gomez et al., 2014, 2015). The above-mentioned studies as well as our observations point to a probable reason why players from category B had significantly higher HR parameters.

### Practical Implications, Limitations and Recommendations for Future Studies

Heart rate observation during games could be helpful for coaches, as they can determine HRR zones in which players should exercise during training sessions to be better prepared for elite wheelchair basketball games. HR in wheelchair basketball exercises should be understood as a physical fitness indicator rather than a marker of fatigue or performance (Coutts et al., 2018; Schneider et al., 2018) because we did not collect information about fatigue (e.g., Rating of Perceived Exertion scale points) and performance in our study. High-intensity training sessions may benefit wheelchair basketball players because of high HRR observed in the current study and time spent in zones IV and V. The intermittent nature of wheelchair basketball was confirmed (all the players were observed in all HR zones, even though they spent most of the time in zones IV and V). In all analyses among wheelchair basketball players, researchers should divide players at least into two functional categories according to the classification in wheelchair basketball and consider differences in HR responses between players from category A and B. Hydration of players should be taken into account in the future analyses because hypohydration can increase HR during training in running basketball (Berkelmans et al., 2018), and hydration was not considered in the current study. It is recommended that more global analyses should be carried out in order to examine the players’ classification in regard to their percentage contribution in playing time, tournament level (friendly matches, championships), game quarters, final game outcome and final point differential. The players’ hydration and diet should be taken into account in future studies. Further analyses should be conducted on a larger sample group. We recommend analyzing HR in small-sided wheelchair basketball games to identify optimal training approaches to prepare wheelchair basketball players for competition.

## Conclusion

The intermittent nature of wheelchair basketball was confirmed. Wheelchair basketball players from category A and B performed in all HR zones during a game, and they spent most of the time in zones IV and V (80–89% and 90–100% of HR_peak_). It is recommended that more global analyses should be carried out in order to examine the players’ classification in regard to their percentage contribution in playing time, tournament level (friendly matches, championships), game quarters, final game outcome and final point differential. The players’ hydration and proper diet should be taken into account in future studies. Further analyses should be conducted on a larger sample group in order to plan a proper schedule and types of exercises in training sessions.

## Author Contributions

JM devised the structure of the paper, drafted the manuscript, collected and analyzed the data, and commented on the final version. KG and AK collected and analyzed the data, and commented on the final version. NM-A and AM collected and analyzed the data. BM devised the structure of the manuscript, drafted the manuscript, oversaw the whole research process, collected and analyzed the data, and commented on the final version.

## Conflict of Interest Statement

The authors declare that the research was conducted in the absence of any commercial or financial relationships that could be construed as a potential conflict of interest.
